# Intermediate-Term Oncological, Anastomotic and Nutritional Outcomes of the Sutureless L-Shaped Esophagojejunostomy with Endoscopic Assistance (SLEJ) in Totally Laparoscopic Total Gastrectomy: A Follow-Up Analysis of This Technique

**DOI:** 10.3390/medicina62091685

**Published:** 2026-09-02

**Authors:** Sevket Baris Morkavuk, Sumeyra Guler, Ibrahim Burak Bahcecioglu, Mujdat Turan, Gokhan Giray Akgul, Kubilay Kenan Ozluk, Erdi Aydin, Kahraman Dinler, Cagdas Karaman, Mehmet Ali Gulcelik

**Affiliations:** 1 Department of Surgical Oncology, Ankara Gülhane Research and Training Hospital, Faculty of Medicine, University of Health Sciences, Ankara 06018, Turkey; guler.sumeyra@yahoo.com (S.G.); dr.ibb@hotmail.com (I.B.B.); girayakgul@hotmail.com (G.G.A.); drkenanozluk@gmail.com (K.K.O.); erdiaydin27@gmail.com (E.A.); kahramandinler.78@gmail.com (K.D.); dr.cagdaskaraman@gmail.com (C.K.); mehmetali.gulcelik@sbu.edu.tr (M.A.G.); 2 Department of General Surgery, Ankara Gülhane Research and Training Hospital, Faculty of Medicine, University of Health Sciences, Ankara 06018, Turkey; mujdatturan84@hotmail.com

**Keywords:** anastomosis complication-free survival, entry hole, linear stapler, intra-corporeal anastomosis, disease-free survival

## Abstract

*Background and Objectives*: Intra-corporeal esophagojejunostomy after totally laparoscopic total gastrectomy (TLTG) remains one of the most technically demanding steps of minimally invasive gastric surgery. The management of the common entry hole in linear stapler-based reconstructions still relies on advanced intra-corporeal suturing. The sutureless L-shape esophagojejunostomy with endoscopic assistance (SLEJ) technique, previously described by our group, was developed to overcome this limitation by combining an L-shaped linear stapler configuration with intraoperative endoscopic quality control. Building upon our initial results on the perioperative feasibility of the technique, the present study aimed to evaluate its intermediate-term anastomotic, functional, and oncological outcomes. *Materials and Methods*: Patients who underwent TLTG with D2 lymph node dissection and SLEJ reconstruction for gastric cancer between July 2024 and January 2026 were evaluated. Eligibility criteria included a minimum postoperative follow-up of six months, clinical and endoscopic surveillance, and complete contrast-enhanced thoraco-abdominopelvic computed tomography records. Protocol-based upper gastrointestinal endoscopy was performed at six-month intervals irrespective of symptoms to objectively assess anastomotic lumen width, mucosal healing, reflux findings, and possible intraluminal recurrence. The primary endpoint was anastomosis complication-free survival (anastomotic stenosis, alkaline reflux/reflux esophagitis, marginal ulcer, bleeding due to ulceration, and intraluminal recurrence); secondary endpoints were disease-free survival (DFS), local recurrence and changes to nutritional status. *Results*: A total of 26 patients (18 men and 8 women; mean age 59.0 ± 7.8 years) were analyzed. Anastomotic stricture developed in two patients and was successfully managed with two sessions of endoscopic balloon dilation in both. Similarly, alkaline reflux was documented in two additional patients and resolved under medical treatment. None of the patients required surgical revision. The mean ACFS follow-up duration was 12.42 months with ACFS rates of 92.3% at six months and 81.1% from the twelfth month onward. No local anastomotic recurrence was detected during follow-up. Disease progression occurred in four patients (15.4%), presenting as distant organ metastasis (n = 2) or peritoneal carcinomatosis (n = 2). The mean DFS follow-up duration was 12.62 ± 6.18 months, and the twelve-month OS and DFS rates were 82.1% and 84.0%. Postoperative body weight decreased significantly compared with preoperative values (76.69 ± 15.59 kg vs. 63.23 ± 10.55 kg; *p* < 0.001). A significant decrease was observed in the mean SMI between the preoperative and follow-up assessments (*p* < 0.001). The mean preoperative SMI was 49.90 ± 9.04 cm^2^/m^2^, compared with 44.53 ± 7.98 cm^2^/m^2^ at follow-up. No statistically significant changes were observed between the preoperative and follow-up periods for serum albumin and PNI variables (*p* = 0.703 and *p* = 0.970). *Conclusions*: The present intermediate-term analysis suggests that the SLEJ technique is feasible and associated with acceptable intermediate-term anastomotic, functional, and oncological outcomes in this preliminary single-center cohort. By standardizing common entry-hole management without intra-corporeal suturing and incorporating intraoperative endoscopic quality control, the technique offers a feasible alternative to established linear stapler-based reconstructions.

## 1. Introduction

Laparoscopic total gastrectomy (LTG) was first described by Azagra JS. et al. in 1996. LTG gained acceptance in clinical practice much later than minimally invasive distal gastrectomy due to concerns regarding the laparoscopic feasibility of D2 lymph node dissection and the high technical complexity of intra-corporeal esophagojejunostomy [1]. One of the most significant technical challenges impeding the widespread adoption of TLTG is the establishment of a reproducible and standardized intra-corporeal esophagojejunostomy method between the esophagus and jejunum that is safe, functional, and compliant with oncological principles. In line with this, driven by the widespread adoption of TLTG, various reconstruction methods primarily based on circular and linear staplers have been developed to achieve a safe and reproducible intra-corporeal esophagojejunostomy. Both approaches offer unique advantages in surgical feasibility, anastomotic geometry, lumen width and technical requirements. However, a gold-standard technique capable of simultaneously providing all the characteristics required of an ideal esophagojejunostomy method, namely, safety, a wide anastomotic lumen width, technical simplicity and adaptability to diverse patient anatomies, has not yet been established. While anastomotic leakage rates following intra-corporeal esophagojejunostomy are reported to be between 1.5% and 8% in current series, anastomotic stricture rates can reach up to 15% depending on the stapler configuration used [2,3,4,5].

Circular stapler-based techniques (transoral anvil and intra-corporeal purse-string) have been used for many years in reconstruction after total gastrectomy, adapting the end-to-side anastomotic geometry of open surgery to minimally invasive surgery. However, the risk of esophageal or pharyngeal injury during transoral anvil placement, feasibility issues in patients with limited mouth opening or anatomical constraints, and the requirement for an intra-corporeal purse-string suture represent the main technical challenges of this method [3,6]. Furthermore, there is a risk of developing early postoperative stenosis in patients with a narrow esophageal lumen diameter. These outcomes have paved the way for the development of linear stapler-based esophagojejunostomy techniques, which possess the potential to create a wider anastomotic lumen. The overlap technique, described by Inaba et al. as one of the most common examples of these approaches, allows for the creation of a larger-diameter anastomosis by utilizing a linear stapler in a side-to-side configuration. The main advantages of the overlap technique include its ability to form a wide anastomotic lumen, its more balanced distribution of tension across the anastomotic line, and its applicability through appropriate technical modifications despite the limited mobility of the distal esophagus [7]. However, closure of the common entry hole remains one of the most critical and technically demanding steps of this technique. Closing this opening via intra-corporeal suturing or additional stapler firing not only requires advanced laparoscopic experience due to the limited working space around the subdiaphragmatic area and the hiatus but can also complicate the standardization of the technique by impacting the anastomotic geometry and luminal patency during the closure process. Following the description of the overlap technique, various linear stapler modifications such as the π-shaped, semi-overlap and functional end-to-end techniques have been developed to reduce tension on the anastomotic line, preserve luminal width and increase the safety of the reconstruction. Nevertheless, the vast majority of these modifications continue to rely on the secure closure of the common entry hole and require advanced intra-corporeal reconstruction skills, much like the original overlap technique [7,8,9].

Based on this requirement, the sutureless L-shaped esophagojejunostomy with endoscopic assistance (SLEJ) technique was developed in our clinic to eliminate the need for intra-corporeal suturing and manage the common entry hole through a more standardized approach. In this technique, the esophagojejunostomy is created using an L-shaped configuration of the linear stapler; the common entry hole is closed without the use of intra-corporeal suturing; and this critical phase is performed under simultaneous endoscopic guidance. Through endoscopic assessment, we aim to verify the anastomotic lumen width and the placement of the stapler line and mucosal integrity, thereby enhancing the safety of reconstruction and preserving anastomotic geometry. Consequently, this approach aims to technically simplify reconstruction, making the procedure more standardized and reproducible across different anatomical conditions. The surgical steps of the SLEJ technique, alongside its early-term feasibility and safety outcomes, have been previously detailed in our published study [6].

The purpose of this study is to evaluate the intermediate clinical outcomes of the SLEJ technique, the technical description and early feasibility of which were previously reported in our cohort. In this context, the objectives are to assess the intermediate oncological outcomes, anastomotic safety, and nutritional results of this technique, which represent key determinants of functional recovery after total gastrectomy in TLTG cases. We also aim to demonstrate the safety and feasibility of the technique through systematic clinical and endoscopic follow-up data.

## 2. Materials and Methods

### 2.1. Study Population

This study is the second-phase analysis of a single-center ambispective cohort designed to evaluate the intermediate-term oncological, functional, and nutritional outcomes of the sutureless L-shaped endoscopy-assisted esophagojejunostomy (SLEJ) technique. We reported the early technical and perioperative results for this in a past study. The study included patients who underwent totally laparoscopic total gastrectomy (TLTG) with D2 lymph node dissection for gastric cancer and received an esophagojejunostomy using the technique we termed SLEJ anastomosis at the Surgical Oncology Clinic of Gulhane Training and Research Hospital between July 2024 and January 2026. The inclusion criteria were as follows: a minimum postoperative period of 6 months, follow-up maintained at our institution, endoscopic evaluation performed by our team, and the completion of a contrast-enhanced (oral and intravenous) thoraco-abdominopelvic computed tomography (CT) scan to investigate local recurrence and/or distant organ metastasis. Patients who underwent open surgery, those who received an esophagojejunostomy using a different reconstruction technique, and those with incomplete or inaccessible follow-up data were excluded from the study. During this period, 38 patients were identified who underwent laparoscopic total gastrectomy with esophagojejunostomy using the SLEJ technique. Of these, 12 patients were excluded from the study: 8 because their surgeries were performed within the past 6 months, 2 because they declined the endoscopic evaluation, and 2 due to inaccessible medical oncology and CT records from external centers. Ultimately, a total of 26 patients were included in the study. This study was approved by the Scientific Research and Evaluation Board of the University of Health Sciences, Gulhane Training and Research Hospital (Approval number: 03.03.2026/4-31).

### 2.2. Study Design

In this analysis, a total of 26 patients were evaluated by including the 21 patients analyzed in our previous technical description paper along with five additional patients who were operated on under the same study protocol and met the minimum six-month follow-up criteria. The operations of both the patients in the previous cohort and the newly included patients were performed by the same surgical team. Following TLTG and D2 lymph node dissection, esophagojejunostomy reconstruction was performed using the SLEJ technique. The surgical technical details regarding TLTG, D2 lymph node dissection, and the SLEJ procedure are not repeated here as they are described in detail in our previously published study. The patients were followed up to evaluate the intermediate-term oncological and functional outcomes of the SLEJ technique in addition to the standard oncological follow-up protocol applied for gastric cancer. Patients were scheduled for outpatient clinic visits at 3-month intervals during the first postoperative year and at 6-month intervals thereafter. Physical examination, body weight, routine biochemical analyses, complete blood count, and serum tumor markers were evaluated during the follow-up period. Patients were also advised to present immediately without waiting for their scheduled appointment times in the event of developing dysphagia, difficulty with oral intake, reflux symptoms, or gastrointestinal tract complaints that could be associated with the anastomosis. Oral and intravenous contrast-enhanced thoraco-abdominopelvic computed tomography was performed at postoperative month 6 to screen for local recurrence and/or distant organ metastasis. In patients with a suspicious elevation in serum tumor markers or clinical suspicion of recurrence during follow-up, the timing of imaging was brought forward based on clinical necessity. To evaluate the intermediate-term safety of the SLEJ anastomosis, upper gastrointestinal endoscopy was performed on all patients as part of the study protocol. Through endoscopic assessment, the anastomotic lumen width, integrity of the anastomotic line, mucosal healing status, characteristics of the stapler line, and signs of alkaline reflux and reflux esophagitis were evaluated. Additionally, intraluminal mucosal recurrence at the anastomotic site and other potential pathological findings were recorded. Regardless of the presence of symptoms, endoscopic evaluation was performed at six-month intervals during the first postoperative year. However, in patients presenting with dysphagia, difficulty with oral intake, or reflux symptoms, additional evaluations were carried out based on clinical necessity. Anastomotic stricture was defined as a luminal narrowing that did not permit the passage of a standard adult upper gastrointestinal endoscope through the esophagojejunostomy anastomosis. Findings of alkaline reflux and reflux esophagitis were recorded during the endoscopic assessment and confirmed histopathologically.

The patients’ pathology reports were analyzed. The histological type, degree of differentiation, proximal and distal surgical margins (R0/R1 resection), the numbers of lymph nodes dissected, and those with metastases were recorded. TNM staging of gastric adenocarcinoma was performed in accordance with the 8th edition of the AJCC guidelines, and gastric neuroendocrine neoplasms were staged using the separate 8th edition of the AJCC section for gastric neuroendocrine tumors [10]. The primary endpoint of the study is evidence of intermediate-term anastomotic complications rather than oncological outcomes. The reconstruction performed was identical irrespective of tumor histology, and all 26 patients were included in the anastomotic analyses. To evaluate nutritional outcomes, the patients’ preoperatively recorded body weights were compared with the weights measured during the postoperative follow-up period to assess total weight loss as percentages. Changes in skeletal muscle index (SMI) between the preoperative and postoperative periods were evaluated by reviewing thoraco-abdominal CT images obtained for metastatic staging before surgery and during postoperative follow-up. When preoperative CT examinations were performed at external institutions, patients were contacted individually so that the original images could be retrieved and uploaded to our hospital’s imaging system for standardized reassessment. Postoperative CT studies obtained at approximately 6 months were preferentially selected. Skeletal muscle was manually segmented on a single 1 mm axial CT slice at the third lumbar vertebral level with Core-slicer 1.0, using a −29 to +150 Hounsfield unit threshold. SMI was calculated as skeletal muscle area divided by height squared (cm^2^/m^2^) [11]. To ensure homogeneity, all segmentations were performed by a single investigator who was blinded to clinical outcomes and endoscopic findings at the time of postoperative month 6. Preoperative and postoperative values were then compared to characterize changes in skeletal muscle status following gastrectomy.

In parallel, serum albumin levels and total lymphocyte counts obtained as part of routine laboratory work-up were used to calculate the Prognostic Nutritional Index (PNI) at both time points using the formula PNI = 10 × serum albumin (g/dL) + 0.005 × total lymphocyte count (/mm^3^) [12]. For the preoperative value, the laboratory result obtained within 1 week before surgery and closest to the operation date was used, while postoperative PNI was derived from parameters obtained during the corresponding follow-up assessment. To ensure that the measurements compared were obtained within comparable time frames, all postoperative nutritional variables (body weight, BMI, SMI, serum albumin, and PNI) were derived from the postoperative assessment conducted at month 6, as defined in the protocol.

### 2.3. Definition of Endpoints

The primary endpoint of our study is late anastomosis complication-free survival (ACFS). ACFS is defined as (i) anastomotic stricture, defined as the inability of a standard adult gastroscope to pass through the anastomosis and/or the need for endoscopic balloon dilation; (ii) alkaline reflux/reflux oesophagitis requiring pharmacological treatment, confirmed endoscopically and histopathologically; (iii) marginal ulcer at the anastomosis due to alkaline reflux oesophagitis; (iv) bleeding due to ulceration; and (v) intraluminal (mucosal) recurrence along the anastomotic line. The ACFS evaluation was based on anastomotic complications that developed after the first 30 days. Patients who were alive and event-free upon last contact were censored at that date. Anastomotic leaks, perforations, and anastomotic hemorrhages prior to discharge and classified as anastomotic complications in our first article were not included in the second phase of our study. These were excluded from the second-phase study as they were fully reported in the technical description of the first cohort and follow a biological course distinct from the fibrotic and inflammatory processes underlying late stricture and reflux disease. A review of the literature reveals that the biology of anastomotic stenosis develops in seven stages: anastomosis → inflammation → granulation → collagen deposition → fibrosis → contraction/remodeling → clinical stenosis. This process varies between a median of 43 and 58 days; thus, we included events occurring 30 days later within the scope of ACFS [13]. Early-term complications were not counted again in the second-phase analysis, but they are not disregarded. Consequently, the patient this applies to is included in the statistics for both early- and late-term complications. As a result, the possibility of a causal link between the early-term event and the subsequent stenosis cannot be dismissed.

In this study, the fundamental objective in evaluating the SLEJ technique is to assess the intermediate-term reliability and functional outcomes of esophagojejunostomy reconstruction performed after TLTG, executed in accordance with standard oncological principles. Therefore, the primary endpoint was designated as late ACFS to evaluate the development of technique-specific, reconstruction-related complications over time. Although the oncological efficacy of the technique was not the primary objective of the study, it was analyzed to evaluate how the surgeries performed comply with current guidelines and reference outcomes reported from high-volume centers. Consequently, the secondary endpoints of the study are disease-free survival (DFS) and the development of local recurrence and nutritional status. However, we would like to highlight an important note here. In the OS and DFS analyses, only the 21 patients who were diagnosed with adenocarcinoma were included in the statistical analysis. Neuroendocrine tumors and adenocarcinomas have different mean overall survival and disease-free survival rates. We adopted this approach to minimize heterogeneity.

### 2.4. Statistical Analysis

Statistical analysis was performed using SPSS v22.0 software (SPSS Inc., Chicago, IL, USA). The distribution and normality of continuous variables were evaluated using the Kolmogorov–Smirnov and Shapiro–Wilk tests. Normally distributed continuous variables are presented as mean ± standard deviation (SD), non-normally distributed continuous variables are presented as the median (range), and categorical variables are presented as numbers (n) and percentages (%). When comparing related continuous variables between the preoperative and postoperative periods, a paired samples *t*-test was used for parametric variables, and the Wilcoxon Signed-Rank test was used for non-parametric variables. ACFS, OS and DFS analyses were performed using the Kaplan–Meier method and 95% confidence intervals (CI) were computed using Greenwood’s formula at 6, 12, 18 and 22 months; the number of patients at risk during each time point is displayed under the survival curves. The statistical significance level was set at *p* < 0.05.

## 3. Results

### 3.1. Patient Characteristics

Out of 38 patients who underwent esophagojejunostomy using the SLEJ technique, the outcomes of 26 patients who met the study criteria were analyzed. Of these patients, 18 were male (69.2%) and 8 were female (30.8%). The mean age was 59.0 ± 7.8 years, with a mean age of 59.4 years in male patients and 58.0 years in female patients. Among the patients included in the study, Siewert type II and type III tumors were identified in 14 and 12 patients. Neoadjuvant treatment had been administered in most cases (76.9%). Upon histopathological evaluation, classical-type adenocarcinoma emerged as the predominant subtype, accounting for 16 patients, whereas grade 3 (poorly differentiated) tumors were observed in nine cases. With respect to pathological T stage, 10 patients presented with T1 disease and seven had T4 disease. The mean number of dissected lymph nodes was 24.85, and the mean number of metastatic lymph nodes was 3.15. A tumor-free surgical margin was achieved in all patients, with cases of R1 or R2 resection. The mean preoperative body weight was 76.69 kg, whereas the mean body weight measured during the postoperative follow-up period was 63.23 kg. The mean body weight loss was 13.46 kg; this value was 14.67 kg in male patients and 10.75 kg in female patients. Preoperative laboratory evaluation showed a mean serum albumin level of 4.08 g/dL, compared with 4.06 g/dL at follow-up. The mean preoperative Prognostic Nutritional Index (PNI), skeletal muscle index (SMI) and body mass index (BMI) were 50.56, 49.90 cm^2^/m^2^ and 27.12 kg/m^2^, respectively. At follow-up, the corresponding mean values were 49.59, 44.53 cm^2^/m^2^ and 22.45 kg/m^2^. No local recurrence was detected in any patient during the follow-up period. Disease progression developed in four patients (15.4%); distant organ metastasis was identified in two of these cases (one liver and one lung metastasis), while peritoneal carcinomatosis was detected in the remaining two cases. When tumor histopathology was evaluated, a signet ring cell component was present in three of the four patients, while grade 3 differentiation was observed in one patient. No local recurrence or distant metastasis developed in 22 patients (84.6%) during the follow-up period. The mean DFS follow-up duration was 13.19 months, and the overall survival follow-up duration was 13.73 months for all patients without excluding those for pathological diagnoses. Three patients (11.5%) died during the follow-up period. While distant organ metastasis was present in two of these patients, one patient died due to causes unrelated to gastric cancer (Table 1).

### 3.2. Intermediate-Term Functional Outcomes of the SLEJ Technique

The intermediate-term functional and anastomotic outcomes of the SLEJ technique were evaluated via clinical follow-up and upper gastrointestinal endoscopies performed as part of the study protocol. In the endoscopic evaluations conducted at six-month intervals, no anastomosis-related complications and/or intraluminal recurrence were detected in 22 out of 26 patients (84.6%). We observed stricture at the esophagojejunostomy anastomosis in two patients describing dysphagia with solid food intake, and findings compatible with alkaline reflux were found in two patients presenting with complaints of chest pain and heartburn. The mean ACFS follow-up duration was 12.42 months (Table 2).

The first patient who developed anastomotic stricture was the case in whom postoperative anastomotic dehiscence had been reported in our initial study. In this patient, the anastomosis had been safely completed using an additional stapler, and minimal extraluminal contrast leakage was detected upon oral and intravenous contrast-enhanced computed tomography. This was performed due to discoloration observed in the drain at the esophagojejunostomy site during the postoperative period. The patient, who was followed up with conservative treatment, underwent an upper gastrointestinal endoscopy due to the development of dysphagia and a sensation of solid food catching at the ninth postoperative month following discharge. The endoscopic assessment revealed a stricture that did not permit the passage of a standard adult endoscope through the anastomosis. Following two sessions of endoscopic balloon dilation performed one week apart, complete clinical improvement was achieved without the need for additional surgical intervention. The patient’s last follow-up examination was completed at month 21 of the postoperative period. During this period, there were no reports of intraluminal recurrence, anastomotic stricture, stenosis, bleeding, ulceration, or alkaline reflux esophagitis. An upper gastrointestinal endoscopy was performed on a patient who described dysphagia symptoms in the tenth postoperative month, revealing a stricture that made it difficult for a standard adult endoscope to pass through the anastomosis. Following two sessions of endoscopic balloon dilation performed one week apart, complete clinical improvement was achieved without the need for additional surgical intervention, similar to the first patient. The patient’s medical history revealed that no complications had developed during the gastrectomy or SLEJ reconstruction phases. Furthermore, no findings suggestive of anastomotic stricture were detected during the follow-up endoscopy at 6 months postoperatively. The patient’s last follow-up examination was completed at month 20 in the postoperative period. During this period, there were no reports of intraluminal recurrence, anastomotic stricture, stenosis, bleeding, ulceration, or alkaline reflux esophagitis. For the third patient who described chest pain and heartburn complaints at postoperative month 5, endoscopic evaluation revealed mucosal edema, hyperemia in the distal esophagus, and bile reflux. The diagnosis of alkaline reflux was confirmed through histopathological evaluation, which identified reactive changes compatible with reflux esophagitis. The patient was managed conservatively with ursodeoxycholic acid, sucralfate, and prokinetic agents. No surgical intervention was required, and the patient’s medical history indicated that no complications related to the surgery developed. A standard gastrectomy with SLEJ reconstruction was performed. The patient’s last follow-up was 10 months postoperatively, and the patient passed away the same week due to non-oncological reasons. For the last patient who described chest pain and heartburn complaints at postoperative month 6, endoscopic evaluation revealed mucosal edema, hyperemia in the distal esophagus, and bile reflux. The patient’s medical history indicated no complications related to the surgery, and a standard gastrectomy with SLEJ reconstruction had been performed. The patient was managed conservatively with ursodeoxycholic acid, sucralfate, and prokinetic agents. No surgical intervention was required. The patient’s last follow-up examination was completed at month 13 of the postoperative period. During this period, no reports of intraluminal recurrence, anastomotic stricture, stenosis, bleeding, ulceration, or alkaline reflux esophagitis were made (Table 3).

**Figure 1:**
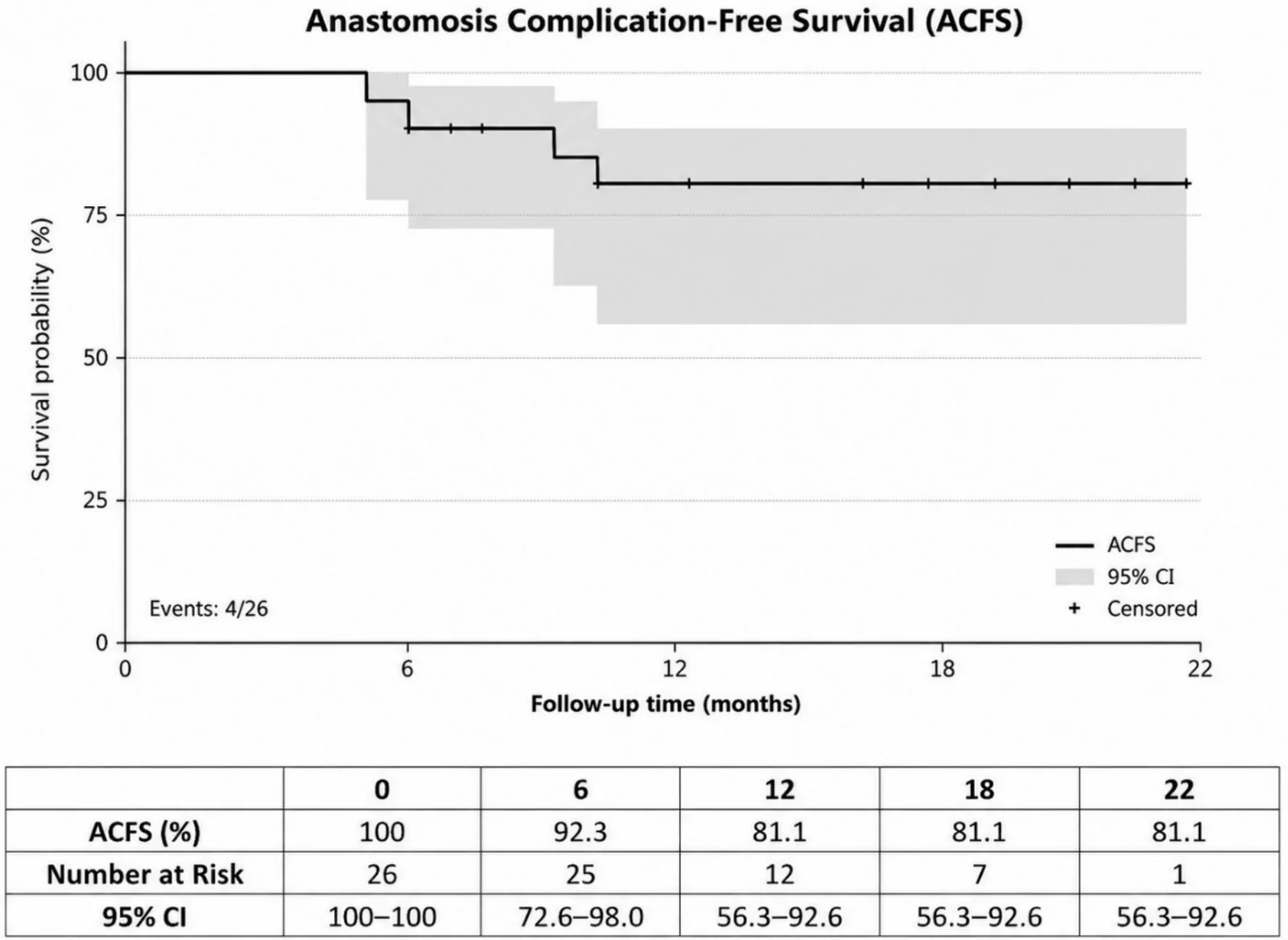
Intermediate-term functional strength of SLEJ reconstruction: Kaplan–Meier analysis of anastomotic complication-free survival (ACFS).

Late ACFS analysis was performed using the Kaplan–Meier method. The Kaplan–Meier curve demonstrated that anastomotic complications clustered within the first 10 months. We observed that while the number of patients at risk decreased during the subsequent follow-up intervals, the confidence interval widened accordingly. From month 10 onward, the survival curve plateaus and remains stable at 81.1%. ACFS starts at 100%, declining to 92.3% (95% CI 72.6–98.0) at month 6 and to 81.1% (95% CI 56.3–92.6) at month 12. This level remains constant and unchanged at months 18 and 22. The number of patients at risk was 26 at the beginning, 25 at month 6, 12 at month 12, 7 at month 18, and 1 at month 22 (Figure 1). At the prespecified 22-month follow-up period, the restricted mean complication-free survival time was 19.33 months (95% CI, 16.73–21.38) for ACFS.

### 3.3. Evaluation of Nutritional Status and Tumor Biomarkers

The efficacy of our SLEJ anastomotic technique on postoperative nutritional balance and tumor biomarkers was investigated. During the patients’ preoperative and follow-up period, weight tracking, albumin levels, and BMI, SMI, and PNI ratios were analyzed for nutritional status, and CEA was used for tumor biomarker analysis. Prior to statistical analysis, variables were subjected to homogeneity and distribution tests. It was found that the change in body weight, SMI, and BMI showed a homogeneous distribution, whereas changes in carcinoembryonic antigen, albumin, and PNI did not display a homogeneous distribution. When comparing preoperative and postoperative body weights, we found a statistically significant decrease (paired samples *t*-test, *p* < 0.001). The mean body weight was 76.69 ± 15.59 kg in the preoperative period and 63.23 ± 10.55 kg during the postoperative follow-up period. The mean body weight loss was 13.46 kg, corresponding to approximately 17.6% of the initial body weight. Mean BMI and SMI values differ significantly between the preoperative and follow-up periods. Paired-samples *t*-test analysis showed that the mean BMI decreased from 27.12 ± 5.35 kg/m^2^ preoperatively to 22.45 ± 4.48 kg/m^2^ at follow-up, and this reduction was statistically significant (*p* < 0.001). Similarly, a significant decrease was observed in mean SMI between the preoperative and follow-up assessments (*p* < 0.001). The mean preoperative SMI was 49.90 ± 9.04 cm^2^/m^2^, compared with 44.53 ± 7.98 cm^2^/m^2^ at follow-up. For serum albumin and PNI, preoperative and follow-up results were compared using the Wilcoxon Signed-Rank test. No statistically significant changes were observed between the two periods. The median values of both parameters were similar between the preoperative and follow-up periods (*p* = 0.703 for albumin and *p* = 0.970 for PNI).

When the entire study population was included in the analysis for CEA, we found no significant difference between the median preoperative CEA level and the median postoperative follow-up CEA levels (*p* = 0.054) according to Wilcoxon’s signed-rank test. The median CEA level was 3.54 ng/mL preoperatively and 2.65 ng/mL during the follow-up period. However, it is impossible to prevent distant organ metastasis or peritoneal carcinomatosis through our technique or any standard gastrectomy techniques accepted in the literature. Therefore, the CEA biochemical follow-up analysis was performed on 22 patients who did not develop metastatic progression, excluding the patients who developed distant organ metastasis (n = 2) and peritoneal carcinomatosis (n = 2). In this patient group, preoperative and postoperative CEA levels were compared using the Wilcoxon Signed-Rank test, and a statistically significant decrease was observed (*p* < 0.001). The median preoperative CEA level was 3.92 ng/mL, and the median postoperative follow-up CEA level was 2.50 ng/mL (Table 4).

### 3.4. Analysis of Oncological Outcomes Using the Kaplan–Meier Test

**Figure 2:**
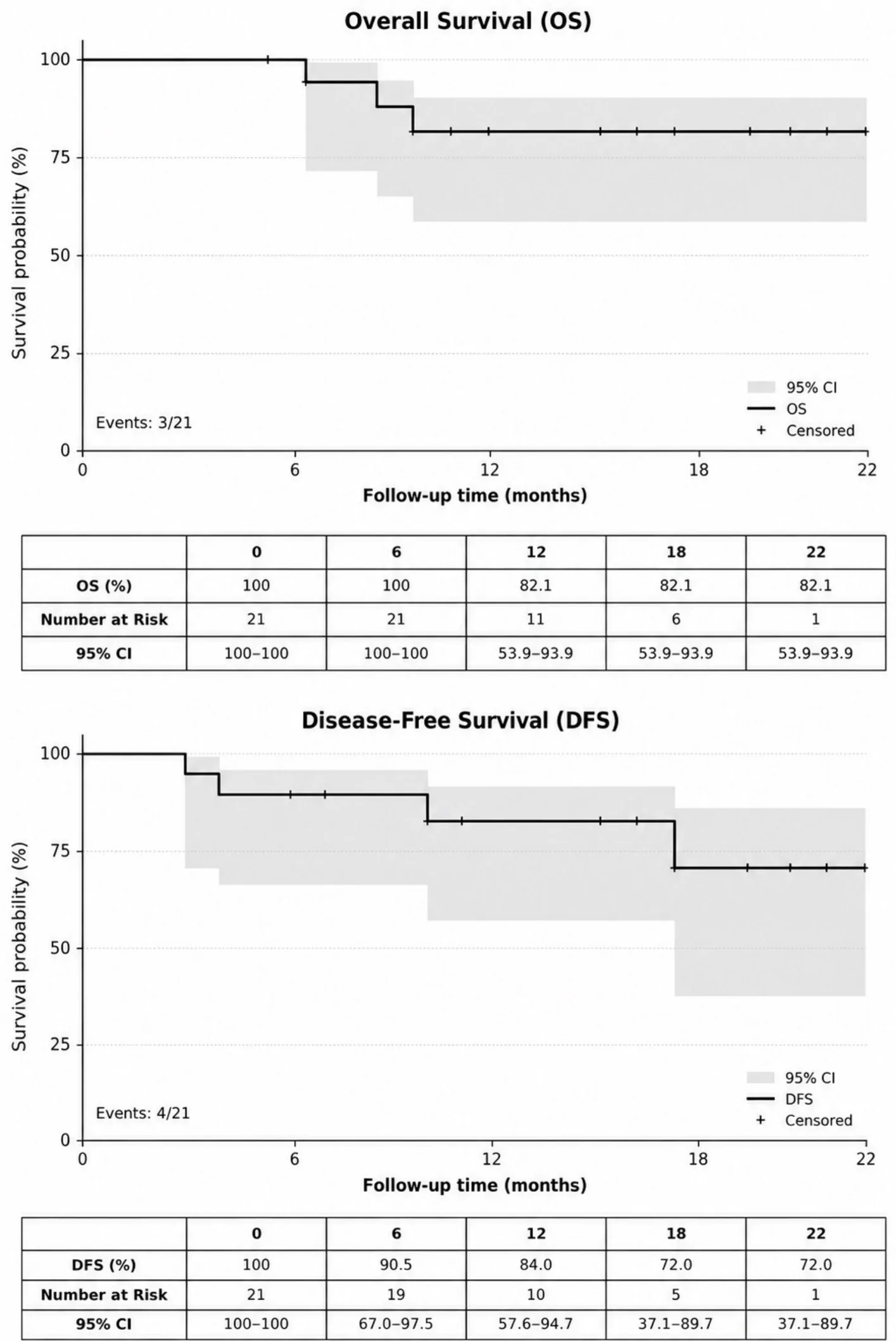
Kaplan–Meier analysis of overall survival and disease-free survival after SLEJ reconstruction.

OS and DFS analyses were performed using the Kaplan–Meier method for only adenocarcinoma pathological cancer patients (n = 21). For mean overall survival, the mean follow-up period of the patients was 13.29 ± 5.75 months (median 12.0, range 6–22 months). During the follow-up period, three patients (14.3%) died, and all mortality events occurred between the 7th and 10th months. Overall survival (OS) was 100% at six months and 82.1% (95% CI: 53.9–93.9) at 12 months, remaining unchanged through months 18 and 22. The number of patients at risk was 21 at the beginning and at postoperative month 6, 11 at month 12, 6 at month 18, and 1 at month 22. At the prespecified 22-month follow-up period, the restricted mean overall survival time was 19.63 months (95% CI, 16.93–22.00) (median OS could not be calculated). DFS was analyzed in all 21 patients using the Kaplan–Meier method; four patients experienced disease progression (distant organ metastasis or peritoneal carcinomatosis) during follow-up. The mean DFS follow-up duration was 12.62 ± 6.18 months (median 11.0, range 3–22 months). Disease progression developed in four patients during the follow-up period. The DFS rate was 90.5% (95% CI 67.0–97.5) at month 6, 84.03% (95% CI 57.6–94.7) at month 12, and 72.03% (95% CI: 37.1–89.7) at month 18. Since no new disease progression was observed after the 17th month during the follow-up period, the DFS rate continued at 72% until the 22nd month. While the initial number of patients at risk was 21, the rate was calculated as 19, 10, 5, and 1 at the 6th, 12th, 18th, and 22nd months, respectively (Figure 2). At the prespecified 22-month follow-up period, the restricted mean disease-free survival time was 18.86 months (95% CI: 15.84–21.29) (median DFS could not be calculated).

## 4. Discussion

Despite the widespread adoption of TLTG, intra-corporeal esophagojejunostomy remains one of the most technically challenging steps of this procedure. In particular, the anatomically limited mobility of the distal esophagus, the narrow working space around the hiatus, and the requirement for intra-corporeal suturing in certain techniques are among the primary factors that increase the experience-dependent nature of performing this stage. Therefore, when evaluating new reconstruction techniques, it is of great importance to demonstrate not only perioperative feasibility and early complication rates but also the mid- and long-term functional performance and oncological safety of the anastomosis. In this study, the intermediate-term clinical outcomes of the SLEJ technique were evaluated in an expanded patient cohort, and its functional and oncological reliability was analyzed. The technical details and early results of this method were previously reported.

Unlike existing linear stapler-based reconstruction methods, the fundamental design of the SLEJ technique eliminates the need for intra-corporeal suturing in common entry-hole management, rather than standardizing it, creating a new anastomotic geometry. In addition, the routine use of endoscopic assistance allows for the intraoperative evaluation of luminal patency and mucosal integrity during the closure of the stapler line. Thus, technical problems that could affect the lumen can be recognized during the operation, and immediate correction is possible when necessary.

Patient series and systematic reviews reported in the literature demonstrate that anastomotic complications after intra-corporeal esophagojejunostomy are largely associated with the reconstruction technique used, stapler configuration, and surgical experience. In reviews and meta-analyses evaluating intra-corporeal esophagojejunostomy techniques, linear stapler-based methods such as overlap, functional end-to-end, π-shaped, and modified π-shaped are generally observed to be performed with low anastomotic leak rates and acceptable stenosis rates. In a meta-analysis by Sozzi et al. involving 3156 patients, the anastomotic leak rate was reported as 2.8% in the linear stapler group and 3.8% in the circular stapler group. As for anastomotic stricture, the rate is 5.3% in techniques utilizing circular staplers and 0.8% in the group encompassing linear stapler anastomotic techniques. This difference was statistically significant, and the use of linear staplers was associated with a significantly lower risk of stenosis [14]. In a meta-analysis by Aiolfi et al. encompassing 3177 patients that compared the overlap, functional end-to-end, SST, HDST, and OrVil techniques, no significant difference was demonstrated in terms of leakage or stenosis [15]. In the meta-analysis by Inokuchi et al., anastomotic leak rates ranged between 1.1% and 3.2%, while anastomotic stricture rates for methods other than OrVil were found to vary between 1.0% and 3.6%. They attributed this outcome to the fact that it is a multifactorial complication not solely dependent on the type of stapler used. Rather, a multitude of surgical variables contribute to this outcome, such as anastomotic geometry, the luminal diameter created, orientation of the stapler line, tissue perfusion, and the closure technique of the common entry hole in particular [3]. When evaluated from this perspective, the SLEJ technique focuses on standardizing common entry-hole management, which is thought to play a critical role in the development of complications rather than developing a new linear stapler variation. In our study, although two patients developed anastomotic stenosis, both cases were successfully treated with two sessions of endoscopic balloon dilatation without the need for surgical revision. A detailed review of these cases showed that one of the patients was followed for suspected anastomotic leakage and underwent reconstruction using one additional stapler. Despite our small number of cases, we attribute our low rate of stenosis to the use of endoscopy to control lumen patency during the anastomosis step. One of the distinguishing features of the SLEJ technique is that endoscopy is utilized for quality control purposes during the creation of the anastomosis itself, rather than solely during postoperative follow-up. The ability to evaluate luminal patency and mucosal integrity immediately after the anastomosis is completed allows potential technical problems to be recognized within the same session. However, due to the limited number of patients and the lack of a direct comparative control group, it is not possible to interpret these results as superior to other linear stapler techniques. These findings suggest that the SLEJ technique may be a safe and feasible alternative to existing linear stapler-based reconstructions.

Alkaline reflux and reflux esophagitis that may develop after esophagojejunostomy may adversely affect the postoperative quality of life of the patient. In addition, it may cause long-term mucosal change and intestinal metaplasia in the anastomosis line. An ideal esophagojejunostomy reconstruction technique should be evaluated not only in terms of its oncological and anastomotic safety but also its anti-reflux performance. In the literature, overlap, functional end-to-end and π-shaped methods were evaluated in terms of reflux frequency, and no significant difference was found between them. These studies agree that technical factors such as anastomosis geometry, orientation of the stapler line, jejunal loop position and Roux-en-Y distal length play a decisive role in the risk of reflux [16]. In this respect, Roux-en-Y esophagojejunostomy is generally accepted as the preferred reconstructive method after total gastrectomy; however, a non-negligible proportion of patients still develop alkaline reflux esophagitis [17]. Temperley et al. reported a series of patients requiring remedial intervention for severe post-gastrectomy alkaline reflux, most of whom underwent reconstruction with a short Roux limb, emphasizing that a Roux limb of adequate length and proper stapler-line orientation are the principal modifiable determinants of anti-reflux performance [18]. Our SLEJ technique uses L-shaped anastomosis of the jejunal loop to the esophagus; the tissue orientation created during closure of the common entry hole and a Roux-en-Y limb length of at least 45–50 cm reduce the risk of reflux of biliopancreatic contents intruding into the esophagus. The successful management of all alkaline reflux cases detected during the follow-up period in our study with medical treatment suggests that the SLEJ technique can offer an acceptable profile in terms of anti-reflux. However, due to both the limited patient population and the diagnosis of alkaline reflux being based predominantly on endoscopic and pathological findings, our current assertion should be regarded as hypothetical. In this regard, our primary objective is to increase the number of patients undergoing anastomosis using the Roux-en-Y technique, extend follow-up periods, and apply advanced objective diagnostic tests such as 24-h ambulatory pH monitoring, 24-h bilirubin monitoring (Bilitec), and hepatobiliary scintigraphy (HIDA) to cases in which symptoms develop.

Weight loss after total gastrectomy does not develop due to the reconstruction technique applied alone. It is a multifactorial process resulting from the combined effects of the complete loss of the gastric reservoir, vagal denervation, accelerated intestinal transit, reduced oral intake, dumping syndrome, and adjuvant treatments. Therefore, postoperative weight loss is accepted as one of the important clinical indicators reflecting the functional adaptation of patients, not the success of the reconstruction technique alone [19]. In our study, an average body weight loss of 13.46 kg (17.6%) was detected during the postoperative follow-up period. This rate can be considered a reflection of the expected physiological process in which multiple factors act together, such as early satiety, hormonal changes, malabsorption, adjuvant treatments, and the biological effects of the disease. In the literature, previous studies have demonstrated that patients may lose approximately 10–20% of their preoperative body weight during the first postoperative year [20]. Therefore, it is thought that the weight loss observed in our study is related to the expected physiological results of total gastrectomy rather than SLEJ reconstruction. For the purposes of comparison, the study by Muneoka et al., which is more appropriate for this analysis, compared circular stapler anastomosis with linear stapler anastomosis following total gastrectomy. They found body weight loss of 15.9 per cent in the circular anastomosis group and 12.6 per cent in the linear stapler group [21]. Assessing weight loss alone as a reference for patients’ nutritional status is an inadequate selection of data. In our study, we performed gastrectomy and lymph node dissection in accordance with standard procedures. We have merely described a new anastomosis technique and do not believe that this will lead to different outcomes in patients’ nutritional status. We therefore compared our patients’ SMI and PNI (albumin–lymphocyte ratio) results to those of other anastomosis techniques reported in the literature. It is well known that the CT-based skeletal muscle index (SMI) is considered an objective measure of skeletal muscle reserve, and low levels are regarded as a predictor of adverse oncological outcomes. There are many studies in the literature examining the relationship between gastric cancer and sarcopenia [22]. In our study, we found that patients’ preoperative SMI level was 49.9 ± 9.04 and their follow-up level was 44.53 ± 7.98. This difference corresponds to approximately 10.77 per cent. In the study by Jiang et al., Billroth II and Roux-en-Y anastomoses were compared following laparoscopic distal gastrectomy. They found a change in SMI of 4.77 per cent in the Billroth II group and 11.89 per cent in the Roux-en-Y group [23]. Sugiyama et al. compared proximal gastrectomy + double tract anastomosis with total gastrectomy + Roux-en-Y anastomosis. They found a mean SMI loss of 9.3% and 18.3%, respectively [24]. Although our sample size was limited, when we compared the results of our study with other anastomosis techniques in the literature, a lower rate of SMI loss was observed with our anastomosis technique. The Prognostic Nutritional Index (PNI) was first developed by Onodera et al. It is an index that combines serum albumin levels with the total lymphocyte count in peripheral blood [12]. Albumin reflects the patient’s protein reserves and the effect of systemic inflammation on nutrition; the lymphocyte count, meanwhile, reflects the cellular immune response and immune competence. Consequently, the PNI is a marker that assesses the host’s nutritional and immunological reserves together rather than simply being a ratio of these two parameters. Tsumura et al. compared proximal gastrectomy + double-flap with total gastrectomy + Roux-en-Y anastomosis. They reported a decline in PNI of 7.3 per cent versus 1.4 per cent at 6 months and 9.8 per cent versus 3.7 per cent at 12 months [25]. A low PNI level indicates a worse prognosis, whilst a high PNI indicates better outcomes. In our study, we found no significant difference between the PNI values in the preoperative and follow-up periods. Notably, PNI remained largely preserved during postoperative follow-up. The mean and median PNI values calculated before surgery remained virtually unchanged during the postoperative recovery period (50.56 ± 3.79 vs. 49.59 ± 6.32 and 51.40 vs. 49.59). No statistically significant difference was found between the preoperative and postoperative periods. We attribute these findings, despite the relatively small sample size, to the single-center nature of this study, the fact that all surgical procedures and postoperative follow-up were performed by the same experienced team, and the routine use of high-protein and immuno-nutrition enteral supplements during follow-up. However, the indirect effects of the reconstruction technique on postoperative nutrition should not be ignored. Anastomotic leakage, clinically significant stenosis, or severe reflux development may delay oral intake and adversely affect nutritional recovery. The successful management of all anastomotic complications developed in our study with endoscopic or medical methods and the fact that no surgical revision was required in any patient suggest that permanent oral intake restriction due to long-term mechanical obstruction did not develop. This observation suggests that the SLEJ technique does not create an additional mechanical barrier that negatively affects the postoperative feeding process.

The main determinants of the risk of local recurrence in gastric cancer are tumor biology, providing R0 resection at surgical margins (technical success), and performing D2 lymphadenectomy in accordance with oncological principles. For this reason, local recurrence data are accepted as an important safety indicator in the evaluation of new reconstruction techniques. In this context, an ideal reconstruction method should not increase the risk of local recurrence or compromise oncological principles. Muneoka et al. compared intra-corporeal and extracorporeal anastomosis methods in their analysis of 1875 patients who underwent laparoscopic gastrectomy with a diagnosis of stage I gastric cancer. Five-year recurrence-free survival rates were reported as 95.6% and 94.3%, respectively [26]. In our study, no local recurrence was observed in the anastomosis line in any of our patients during the follow-up period. This can be considered an important observation in terms of the oncological safety of the SLEJ technique. On the other hand, four patients developed disease progression, two of whom had distant organ metastasis and two had peritoneal carcinomatosis. Histopathological analysis of patients who developed progression showed that they had a signet ring cell component or poor differentiation (grade 3). This finding supports the idea that recurrence is associated with the aggressive biological behavior of the tumor rather than the reconstruction technique applied. According to current guidelines regarding the postoperative follow-up of gastric cancer, CEA and CA 19-9 are the most relevant tumor markers. The Japanese Gastric Cancer Association’s 2025 guideline also recognizes the measurement of CEA and CA 19-9 as particularly useful for follow-up after curative gastrectomy in addition to CT and endoscopy [27]. It notes that, in some patients, a rise in tumor markers may appear approximately 2–3 months prior to the detection of imaging findings. There is no doubt that the use of tumor biomarkers, in conjunction with imaging techniques, plays a significant role in the follow-up of oncology patients for postoperative local recurrence and/or distant organ metastasis. The half-life of CEA is approximately 2–8 days, while that of CA19-9 is 4–8 days. In general practice, four or more half-lives occur in about one month. Following a successful R0 resection, the markers are expected to return to baseline levels [27]. Since some patients were referred to our clinic from other hospitals for treatment and their CA19-9 levels were unavailable in their medical records, we were able to analyze only the CEA marker for biochemical follow-up analysis. We found that there is no statistically significant difference between the median preoperative CEA value and the median postoperative value for all patients included in the study (with and without distant/local metastasis) (3.54 vs. 2.65 ng/mL). However, when excluding the patients who developed distant organ metastasis or peritoneal carcinomatosis from our study, a significant decrease in postoperative CEA levels was observed and compared with preoperative levels (3.92 vs. 2.50 ng/mL). As is well known, CEA is effective in detecting both local recurrence and distant metastasis following gastric cancer. However, this effectiveness alone is not sufficient to rule out the disease. Zhang R et al. evaluated 1708 patients who underwent curative resection for stage I–III gastric adenocarcinoma. They found that CEA levels were significantly higher in patients who developed local recurrence, peritoneal recurrence, or distant metastases compared to those without recurrence. However, they did not find a significant difference in CEA levels among the different types of recurrence [28]. Moriyama J et al. found that 56% of patients who experienced a recurrence were CEA-positive. More importantly, CEA positivity was significantly higher in cases of liver metastasis and lymph node recurrence. In contrast, CA19-9 was found to be more strongly associated with peritoneal recurrence [29]. Lee EC et al. reported that CEA has a sensitivity of 40.6% and a specificity of 89.5% for detecting recurrence during the follow-up of patients with gastric cancer after surgery. In other words, if CEA levels are elevated, we should suspect recurrence; however, if they are normal, we cannot conclude based on CEA alone that there is no recurrence [30]. Although our results appear promising, it is known that a significant proportion of recurrences in gastric adenocarcinoma occur within the first two years; longer follow-up periods are needed to demonstrate long-term safety [31]. For these reasons, these findings should not be interpreted as evidence of long-term oncological efficacy.

Our study has some important strengths. This study evaluates not only the technical applicability of the SLEJ technique but also the intermediate-term clinical results. The fact that all patients were operated on in the same center and by the same surgical team reduces heterogeneity in the surgical technique. The standardized follow-up protocol increased the reliability of the results obtained. In addition, upper gastrointestinal endoscopy was performed within the protocol regardless of symptoms in all patients, allowing the objective evaluation of not only clinically significant complications but also anastomotic lumen width, mucosal healing, reflux findings, and possible intraluminal recurrences. In this respect, our study contributes to the existing literature by providing objective endoscopic follow-up data.

However, our study has some limitations. Most importantly, this includes the single-center observational design, meaning that the number of patients is relatively limited. Secondly, there is no direct control group using overlap, π-shaped, functional end-to-end, or circular stapler anastomosis. Consequently, interpretation of the observed complication and survival rates is limited to comparison with the results of other anastomosis techniques reported in the literature. Thirdly, biochemical surveillance was limited to CEA, as CA 19-9 and CA 72-4 were not consistently requested at baseline. Fourthly, the study design is ambispective rather than fully prospective. The earlier portion of the cohort was identified retrospectively, and although the follow-up protocol was applied uniformly, the completeness and timing of some baseline laboratory data depended on records generated for clinical rather than research purposes.

Despite these limitations, the findings indicate that the previously described SLEJ technique is feasible and associated with acceptable intermediate-term functional and oncological outcomes in this preliminary single-center cohort. Our study provides encouraging outcomes that may support future evaluation of the technique in different centers, larger patient series, and preferably prospective comparative designs.

## 5. Conclusions

This study is a follow-up analysis evaluating the intermediate-term (13.73 ± 5.78, range 6–22 months) oncological and functional safety of the SLEJ technique, the technical description and perioperative and early postoperative outcomes of which have been previously published. The endoscopy-assisted, sutureless L-shaped esophagojejunostomy technique appears feasible and associated with acceptable intermediate-term outcomes. This reconstruction method aims at standardizing the technically challenging steps of intra-corporeal esophagojejunostomy after totally laparoscopic total gastrectomy. In the average thirteen-month follow-up period, anastomosis-related complications were low, and any complications that did develop were successfully managed by endoscopic or medical methods without the need for surgical revision. In most patients, anastomotic integrity and luminal patency were preserved, and the two patients who developed anastomotic stricture were treated with balloon dilation. No local intraluminal recurrence was observed during follow-up. However, the significance of this observation is limited because the majority of recurrences in gastric adenocarcinoma occur within the first two years and the present duration of observation is insufficient to support any claim of long-term oncological safety. However, postoperative weight loss was consistent with the expected physiological process after total gastrectomy and no additional functional disadvantage specific to the SLEJ technique was demonstrated. The distinctive feature of the SLEJ technique is that it standardizes common entry-hole management, which is one of the most critical stages of intra-corporeal esophagojejunostomy, and eliminates the need for intra-corporeal suture at this step. The routine use of intraoperative endoscopic assistance is an important factor supporting the safety of the technique by allowing direct evaluation of anastomotic lumen width and mucosal integrity during the procedure.

The preliminary single-center design, limited number of patients, and short follow-up period limit the generalizability of the results obtained. The findings should be interpreted as supporting the feasible use of this technique. This is based on the acceptable intermediate-term outcomes of SLEJ rather than its superiority over established techniques. Therefore, multicenter studies with larger patient series are needed to determine the potential advantages of this technique by comparing it with other linear- or circular-stapler-based reconstruction methods with feasibility already established in the literature and case series.

## Figures and Tables

**Table 1:** Demographic and clinicopathological distribution of the patients.

**Gender: n (%)**	
Male	18 (69.2%)
Female	8 (30.8%)
**Age, year, mean ± SD, median, range**	59 ± 7.83
	58 (43–76)
**Weight preoperative, kilogram, mean ± SD, median, range**	76.69 ± 15.59
	79.5 (52–116)
**Weight follow-up, kilogram, mean ± SD, median, range**	63.23 ± 10.55
	63 (37–85)
**Weight loss, kilogram, mean ± SD, median, range**	−13.46 ± 13.5
	−13.5 (−47–8)
**Height, centimeter, mean ± SD, median, range**	168.38 ± 8.34
	170 (153–182)
**Tumor location: n (%)**	
Siewert Type 1	0 (0%)
Siewert Type 2	14 (53.8%)
Siewert Type 3	12 (46.2%)
**Tumor Pathology: n (%)**	
Adenocancer classic variant	16 (61.6%)
Adenocancer ring cell variant	5 (19.2%)
Neuroendocrine tumor	5 (19.2%)
**Tumor Differentiation: n (%)**	
Grade 1	10 (38.5%)
Grade 2	7 (26.9%)
Grade 3	9 (34.6%)
**Neoadjuvant Treatment: n (%)**	
Absent	6 (23.1%)
Present	20 (76.9%)
**T stage: n (%)**	
T1	10 (38.5%)
T2	4 (15.4%)
T3	5 (19.2%)
T4	7 (26.9%)
**N stage: n (%)**	
n0	12 (46.2%)
n1	6 (23.1%)
n2	3 (11.5%)
n3	5 (19.2%)
**Lymph node dissection, number, mean ± SD, median, range**	24.85 ± 5.34
	24.5 (14–34)
**Lymph node metastases, number, median, mean ± SD**	3.15 ± 4.62
	1 (0–15)
**Carcinoembryonic antigen preoperative, ng/mL, mean ± SD, median, range**	4.39 ± 3.51
	3.54 (0.8–17.7)
**Carcinoembryonic antigen follow-up, ng/mL, mean ± SD, median, range**	4.17 ± 5.87
	2.65 (0.6–31.8)
**Albumin preoperative, g/dL, mean ± SD, median, range**	4.08 ± 0.28
	4.1 (3.50–4.70)
**Albumin follow-up, g/dL, mean ± SD, median, range**	4.06 ± 0.50
	4.2 (2.70–4.90)
**SMI preoperative, mean ± SD, median, range**	49.9 ± 9.04
	47.43 (36.83–77)
**SMI follow-up, mean ± SD, median, range**	44.53 ± 7.98
	42.61 (33.58–66.6)
**PNI preoperative, mean ± SD, median, range**	50.56 ± 3.79
	51.40 (41.70–56.45)
**PNI follow-up, mean ± SD, median, range**	49.59 ± 6.32
	50.52 (32.90–57.15)
**BMI preoperative, kg/m^2^, mean ± SD, median, range**	27.12 ± 5.35
	26.48 (16.98–35.41)
**BMI follow-up, kg/m^2^, mean ± SD, median, range**	22.45 ± 4.48
	22.06 (14.45–33.62)
**Metastasis: n (%)**	
Absent	22 (84.6%)
Peritoneal carcinomatosis	2 (7.7%)
Lung metastasis	1 (3.85%)
Liver metastasis	1 (3.85%)
**Disease free Survival mean follow-up duration, month, mean ± SD, range**	13.19 ± 6.17
	3–22
**Mortality: n (%)**	
Death	3 (11.5%)
Alive	23 (88.5%)
**Overall Survival mean follow-up duration, month, mean ± SD, range**	13.73 ± 5.78
	6–22

SD, standard deviation; SMI, skeletal muscle index; PNI, prognostic nutritional index; BMI, body mass index.

**Table 2:** Analysis of intermediate-term anastomosis technique complications according to endoscopy check.

**Complication: n (%)**	
Absent	22 (84.6%)
Present	4 (15.4%)
**Anastomotic Complications: n (%)**	
Absent (marginal ulcer, bleeding, intraluminal recurrence)	22 (84.6%)
Anastomotic stenosis	2 (7.7%)
Alkaline reflux- Heartburn	2 (7.7%)
**Anastomotic Complications free Survival, mean follow-up duration, month, mean ± SD, range**	12.42 ± 5.85
	5–22

**Table 3:** Time frames and management of intermediate-term anastomosis complications.

Patient	Complication	Postoperative Month at Diagnosis	Management	Follow-Up, Months
1	Anastomotic stenosis	9	Endoscopic balloon dilatation; no surgical intervention required	21
2	Anastomotic stenosis	10	Endoscopic balloon dilatation; no surgical intervention required	20
3	Alkaline reflux	5	Conservative management with ursodeoxycholic acid; no surgical intervention required	10
4	Alkaline reflux	6	Conservative management with ursodeoxycholic acid; no surgical intervention required	13

**Table 4:** Preoperative and postoperative follow-up changes in nutritional status and carcinoembryonic antigen.

Variable	Patients	Preoperative Analysis	Post-Operative Follow-Up Analysis	Test Statistics	*p* Value
Weight, kilogram, mean ± SD	26	76.69 ± 15.59	63.23 ± 10.55	5.081	*p* < 0.001 ^t^
Skeletal Muscle Index, mean ± SD	26	49.9 ± 9.04	44.53 ± 7.98	8.413	*p* < 0.001 ^t^
Body Mass Index, kg/m^2^, mean ± SD	26	27.12 ± 5.35	22.45 ± 4.48	5.296	*p* < 0.001 ^t^
Albumin, g/dL, median (range)	26	4.1 (3.5–4.7)	4.2 (2.7–4.9)	−0.382	*p* = 0.703 ^Z^
Prognostic Nutritional Index, median (range)	26	51.40 (41.70–56.45)	50.52 (32.90–57.15)	−0.038	*p* = 0.970 ^Z^
Carcinoembryonic antigen, ng/mL, median (range) (all patients)	26	3.54 (0.8–17.7)	2.65 (0.6–31.8)	−1.924	*p* = 0.054 ^Z^
Carcinoembryonic antigen, ng/mL, median (range) (without distant metastasis patients)	22	3.92 (1.1–17.7)	2.50 (0.6–6.5)	−3.598	*p* < 0.001 ^Z^

^t^: Paired-samples *t*-test; ^Z^: Wilcoxon signed-rank test; SD, standard deviation.

## Data Availability

The raw data supporting the conclusions of this article will be made available by the authors on request.

## Abbreviations

**ACFS**, anastomosis complication-free survival; **BMI**, Body Mass Index; **CA 19-9**, carbohydrate antigen 19-9; **CA 72-4**, cancer antigen 72-4; **CEA**, carcinoembryonic antigen; **CI**, confidence interval; **D2**, D2 lymph node dissection; **DFS**, disease-free survival; **EJ**, esophagojejunostomy; **GLOBOCAN**, Global Cancer Observatory; **HDST**, hemi double stapling technique; **IARC**, International Agency for Research on Cancer; **LDG**, laparoscopic distal gastrectomy; **LTG**, laparoscopic total gastrectomy; **OrVil**, transorally inserted anvil delivery system; **OS**, overall survival; **PNI**, prognostic nutritional index; **SD**, standard deviation; **SLEJ**, sutureless L-shaped esophagojejunostomy with endoscopic assistance; **SMI**, Skeletal Muscle Index; **SPSS**, Statistical Package for the Social Sciences; **SST**, single-stapling technique; **TLTG**, totally laparoscopic total gastrectomy.
